# Continuous Microfluidic Purification of DNA Using Magnetophoresis

**DOI:** 10.3390/mi11020187

**Published:** 2020-02-11

**Authors:** Ying Xu, Zhen Zhang, Zhen Su, Xiaoxiang Zhou, Xiaoming Han, Quanjun Liu

**Affiliations:** 1 State Key Laboratory of Bioelectronics, School of Biological Science and Medical Engineering, Southeast University, Nanjing 210096, China; 220174594@seu.edu.cn (Y.X.); 220181842@seu.edu.cn (Z.Z.); 220174564@seu.edu.cn (Z.S.); 230189166@seu.edu.cn (X.Z.); 220171812@seu.edu.cn (X.H.); 2 Shenzhen Research Institute of Southeast University, Shenzhen Virtual University Park, Shenzhen 518000, China

**Keywords:** microfluidics, DNA purification and extraction, magnetophoresis, multi-laminar flow, COMSOL simulation

## Abstract

Automatic microfluidic purification of nucleic acid is predictable to reduce the input of original samples and improve the throughput of library preparation for sequencing. Here, we propose a novel microfluidic system using an external NdFeB magnet to isolate DNA from the polymerase chain reaction (PCR) mixture. The DNA was purified and isolated when the DNA-carrying beads transported to the interface of multi-laminar flow under the influence of magnetic field. Prior to the DNA recovery experiments, COMSOL simulations were carried out to study the relationship between trajectory of beads and magnet positions as well as fluid velocities. Afterwards, the experiments to study the influence of varying velocities and input of samples on the DNA recovery were conducted. Compared to experimental results, the relative error of the final position of beads is less than 10%. The recovery efficiency decreases with increase of input or fluid velocity, and the maximum DNA recovery efficiency is 98.4% with input of l00 ng DNA at fluid velocity of 1.373 mm/s. The results show that simulations significantly reduce the time for parameter adjustment in experiments. In addition, this platform uses a basic two-layer chip to realize automatic DNA isolation without any other liquid switch value or magnet controller.

## 1. Introduction

Purification and isolation of nucleotide samples like deoxyribonucleic acid (DNA) and ribonucleic acid (RNA) is an important step for subsequent characterization and application of various biochemical reaction [1,2,3]. The common methods of purification of DNA include electrophoresis [4], high salt precipitation [5], spin column [6], and magnetic beads [7], etc. Among them, magnetic beads are employed via specific or non-specific adsorption of surface-modified magnetic beads with the corresponding targeted DNA to form new complexes, which are then separated from other non-targeted substances under the influence of magnetic field. Based on the methods in which the magnetic field is created, magnetophoretic separation can be divided into three categories: electromagnet separation [8,9], integrated soft magnet separation [10,11,12], and external permanent magnet separation [13,14,15]. In terms of electromagnet separation, it produces a small magnetic force but flexibly controls the magnitude of the magnetic field. Integrated soft magnets can generate a large magnetic force, but the fabrication procedure of integrated chips is more complex than that of the ordinary two-layer microfluidic chip. In comparison, the external permanent magnet is separately placed beside the chip. This kind of methods to generate magnetic field is cheap and easily accessible, which is ideal for the control of magnetic beads.

Magnetic field combined with microfluidic equipment is expected to realize automatic separation and purification of tiny amount of DNA, which will bring convenience to the application of various molecular biology techniques. According to the previous researches, there are two main methods to extract and purify DNA on the chip via external permanent magnet. The first scheme is to immobilize magnetic beads in the chip and then inject different solutions to bind targeted DNA, wash away the waste, and elute targeted DNA through the same inlet, so complex controller or manual operation is required to change solutions [16,17,18]. Karla et al. [16] used a cyclic olefin copolymer microfluidic device to realize DNA extraction of circulating tumor DNA by firmly capturing the magnetic beads, but it still requires extra operation when changing buffers. The second one is that positions of buffers remain immobilized, and the magnetic beads are controlled to pass through different solutions to complete purification. These buffers can be static or continuous. The movement of beads is controlled through the static buffers by the motion of the external permanent magnet by hand [2,19], while beads cross the continuous flow under the influence of the inhomogeneous magnetic field. Magnetic beads across various continuous flows has been applied in cell sorting [14,20,21], bioanalysis [15,22,23], capsule assembly [24], etc. Kim et al. [25] used “SIM-Chip” that comprised a magnetophoretic separator and a dispenser to enrich circulating tumor cells (CTCs). CTCs can be separated from the mixture and get into running buffer under the influence of magnetic field, but the fabrication of this separator is much more complicated than that of simple two-layer PDMS chip. Karle et al. [26] employed this method to extract DNA, as it can automatically extract and purify DNA without any other manual operation. However, in order to avoid the magnetic beads immobilizing on the channel wall, they used a large circular chip with a rotating magnet regulated by a TMCM-303 controller, which increases the complex of the whole platform and the size of the chip is too big to purify a small amount of rare DNA. In addition, there is no thorough simulation research to study the trajectories of beads in continuous flow separation controlled by permanent magnet. While many parameters such as flow velocities and positions of external permanent magnet have impacts on the movement of beads, our work systematically studies the influence of these parameters on the trajectories of the beads and DNA recovery of magnetic beads.

Here, we demonstrate a novel method of continuous flow separation of nucleic acid sample based on basic branched structure on a polydimethylsiloxane (PDMS) chip without an extra controller for magnet. The traditional magnetic beads purification in test tubes includes three steps: binding, washing, and elution. All these three procedures were transferred into a branched chip. During the first phase of this study, the software COMSOL was utilized for the simulation of the movement of magnetic beads in the channel of complete structure at three positions of permanent magnet and under different flow velocities. These simulations help us find out the appropriate magnet position, where magnetic beads cross the main channel at the maximum path, so the beads can be fully washed. For the reduction of simulation time, we simplify the structure in simulation, which also achieves accurate trajectories of magnetic beads. Finally, we employ microfluidic chip of this structure to isolate the specific fragment of DNA from PCR mixture and calculate DNA recovery efficiency at different input amounts and flow velocities. 

## 2. Working Mechanism

**Figure 1:**
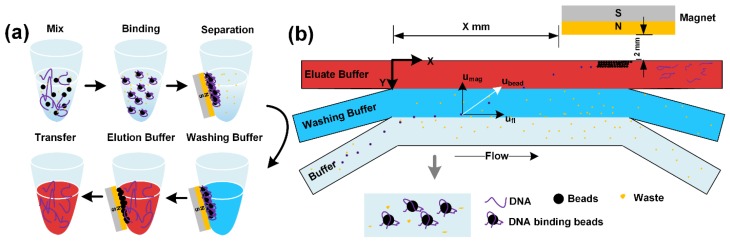
(**a**) Conventional magnetic beads purification of DNA in test tubes. Repeatedly resuspension and adsorption steps of beads are required for purification, leading to the time-consuming and boring experimental operation. (**b**) The schematic for continuous flow separation of magnetic beads to recover DNA via the deflection of DNA-carrying beads across laminar flow streams of washing and elution solutions.

The extraction of DNA from PCR mixture, lysed cells or bacteria requires three steps (Figure 1a). First, DNA is bound to magnetic beads. Then other molecules or waste are removed by washing buffer. Lastly, DNA is eluted from the DNA-carrying beads at the appropriate pH. We design a branched chip to execute these three steps. The chip consists of one main channel connected to three branched channels at both left and right side (ESI Figure S1). The solution of DNA-carrying beads, washing buffer, and the TE buffer are continuously injected into the three inlets, respectively (Figure 1b). These solutions have passed from different branched channels into main channel and formed multi-laminar flow. DNA-carrying beads pass across the interfaces of different buffers via magnetic force provided by external permanent magnet. 

Because of the low Reynolds number, fluid remains in the form of laminar flow. The transport of DNA-carrying magnetic beads could be seen as a result of interaction between magnetic force and drag force on the chip, while other factors including Brownian motion, Van der Waal’s force, and interparticle effects can be neglected in this study considering the concentration and the size of the bead. The beads transport can be predicted by Newton dynamics [27,28,29],

(1) $$m_{bead}\frac{du_{bead}}{dt}=F_{drag}+F_{mag}$$

and

(2) $$F_{drag}=6\pi \eta r\left(u_{fl}-u_{bead}\right)$$

Here $m_{bead},\;u_{bead}$ and r are the mass, velocity, and radius of the beads, while η and $\;u_{fl}$ are the viscosity and velocity of the fluid, respectively. $F_{drag}$ represents the drag force that is defined by Stokes’ law, while $F_{mag}$ represents the magnetic force.

(3) $$F_{mag}=\mu_{0}\Delta \chi V_{bead}\left(H\cdot \nabla \right)H$$

Here $\mu_{0}$ is the permeability of free space, Δχ is the effective magnetic susceptibility of beads, $V_{bead}$ is the volume of beads. H and ∇H are the magnetic field intensity and the gradient of the magnetic field intensity, respectively. The relationship between magnetic field intensity(*H*) and magnetic flux density(B) can be demonstrated by the equation $B=\mu_{0}H$. Then the magnetic force can be rewritten as [30]:

(4) $$F_{mag}=\frac{\Delta \chi V_{bead}\left(B\cdot \nabla \right)B}{\mu_{0}}$$

and the Equation (1) can be rewritten as:

(5) $$\frac{du_{bead}}{dt}=\frac{6\pi \eta r\left(u_{fl}-u_{bead}\right)}{m_{bead}}+\frac{\Delta \chi V_{bead}\left(B\cdot \nabla \right)B}{m_{bead}\mu_{0}}$$

The trajectories of DNA-carrying beads in the channel are simulated using the kinematic equation.

## 3. Materials and Methods

### 3.1. Simulation

The prediction of magnetic beads transport for the proposed system and the magnetic flux distribution of external NdFeB magnets are simulated using COMSOL Multiphysics 5.3a software. The trajectories of magnetic beads are defined on the basis of the model in working machine section. The simulation of complete structure (simulation 1) and main channel of the chip (simulation 2) was done to explain the movement of the magnetic beads (ESI Figure S2). Detailed simulation parameters are shown in the “ESI” Table S1.

### 3.2. Design and Fabrication of the Microfluidics Chip

The structure of the two-dimensional microfluidic chip was designed by the CAD software. The width and height of each separate branched microchannel are 200 μm and 40 μm, respectively, and the width, length, and height of the main microchannel are 600 μm, 10 mm, and 40 μm. The chip is made of PDMS by soft lithography. First, the glass substrate was rinsed in acetone and di-water, and then drought by nitrogen. After that, it was soaked in methanol (34860, Sigma-Aldrich, St. Louis, MO, USA) solution with 5% volume of dimethylchlorosilane (6G-27845, GELEST, INC., Morrisville, PA, USA) for 24 h to be hydrophobic. Enough negative epoxy resists (SU-8 2025, MicroChem Corp, Westborough, MA, USA) was dropped in the wafer and then the wafer was put into spin coater (TT, SÜSS MICROTEC SE, Garching, Germany) at 2000 rpm for 60 s. The processed wafer was baked in vacuum oven at 65 °C for 3 min and then at 95 °C for 3 min. The mask and the wafer were exposed to UV light after setting the appropriate parameters. After baking the wafer again, the developer solution was used to get the pattern. Followed by the preparation of PDMS and curing agent (Sylgard 184 silicone elastomer kit, Dow corning, Midland, MI, USA) in 10:1 ratio and pouring of the mixture on the wafer. The bubbles in mixture were removed and the mixture was solidified using a vacuum oven at 90 °C for 2 h. Followed by the peeling off of the solidified PDMS layer and drilling holes as the inlets and outlets. Lastly, the patterned PDMS and the prepared glass substrate were treated with plasma machine (PDC-002, HARRIC SCIENTIFIC Corp, New York, USA) and bonded together. The blue and red ink were injected into the chip to observe the whole structure (ESI Figure S1).

### 3.3. Sample Preparation

Filtered and purified water (18.2 MΩ cm at 25 °C) was used in all aqueous solutions. In polymerase chain reaction, primer for template plasmid pUC19 (Forward: 5’ caggaaacagctatgac 3’, reverse: 5’ taggcgtatcacgaggc 3’) custom-made by Sangon Biotech, China and polymerase TaKaRa Taq^TM^ (R001AM, Clontech, China) was used. The length of the PCR product is 507 bp. The concentration of DNA is quantified by Qubit 4 fluorometer (Invitrogen, Carlsbad, CA, USA) and Qubit dsDNA HS Assay Kit (Q32851, Invitrogen, USA). The PCR products was characterized by electrophoresis. Agarose, loading buffer for electrophoresis were purchased from Tiangen Biotech Co., LTD, Beijing, China. Samples to be purified were mixed with 1 mL 62 ng/μL PCR fragments and 1.8 mL microbeads (A63880, Beckman Coulter Co., USA), and final concentration of DNA is 22.14 ng/μL. The test bead (TB) solution is prepared using 500 μL PBS buffer (KGB5001, KeyGEN Biotech, Nanjing, China) and 900 μL microbeads. 

### 3.4. On-Chip Experiments

**Trajectories of magnetic beads:** The TB solution, fresh 80% ethanol, and TE buffer (pH 8.0, RNase-free, Ambion™, USA) were injected into three separate inlets at the same velocities via glass syringes (Shanghai Gaoge, China) together with a high-precision injection pump (LSP02-2A, Baoding Longer Precision Pump Co., Ltd, Baoding, China). A 1 cm × 1 cm × 2 cm NdFeB permanent magnet (Ningbo Magnet Co., Ningbo, China) was placed 2 mm away from the main channel in y-direction, where the magnetic field strength is about 307 mT measured by Teslameter (WT10A, Weite Magnetic Technology Co., Ltd, Shangqiu, China). In x-direction, the permanent magnet was placed 1 mm (position 1), 4 mm (position 2), and 7 mm (position 3) from the entrance of the branched channel respectively. At each position, the flow velocities were set from 40 μL/h to 120 μL/h at 20 μL/h intervals. The trajectories of magnetic beads were observed by a stereoscope (SMZ745T, Nikon, Tokyo, Japan) equipped with a high definition CCD camera (U3CMOS, Hangzhou, China). The distance between the final position of beads and the entrance of the main channel was measured by ImageJ freeware (https://imagej.nih.gov).

**DNA recovery:** The DNA-carrying bead solution, fresh 80% ethanol, and TE buffer were injected into three inlets of branched channels at the same velocities. The NdFeB magnet was placed at position 3, and the input amount of DNA to be purified is decided by regulating the volume of the mixture with DNA-carrying beads. To collect the purified DNA and avoid further off-chip elution, we used a 0.5 cm × 0.5 cm × 0.5 cm permanent magnet to bind the connecting pipes between the collecting tube and the outlet. After purification on a chip, DNA was characterized by NanoPhotometer (IMPLEN), Qubit and electrophoresis. Negative control was carried out using TB solution.

## 4. Results and Discussion

### 4.1. The Trajectory of Magnetic Beads

We use simulation to study the movement of the magnetic beads on two chip structures that are represented in “ESI” Figure S2. The simulation 1 utilizes complete structure to fully study the trajectories and final positions of magnetic beads under the influence of different magnetic field and flow velocities. However, it takes a long time to calculate the movement of the beads in the branched channel. In order to reduce the time for simulation, main structure is employed to study the movement and position of magnetic beads. Compared to experimental results, both of simulation data show that the relative error is around 10% at fluid velocity of 1.373 mm/s, while the value of that is less than 5% at other fluid velocities. 

#### 4.1.1. Simulation of Complete Structure 

**Figure 2:**
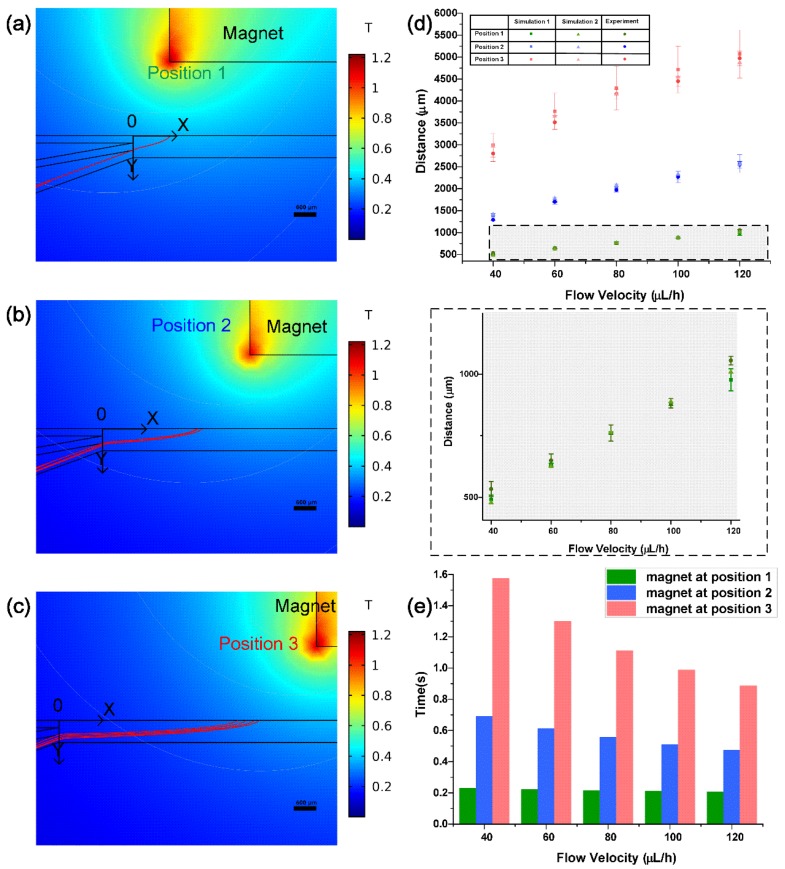
Trajectories of beads with a 1 cm × 1 cm × 2 cm external permanent magnet at different positions in simulation 1(a–c), bar = 600 μm. All the beads (**a**), part of the beads (**b**), and few beads (**c**) deflected to the wall of the branched channel with 𝑢_𝑓𝑙_ = 120 uL/h; (a) *x* = 1 mm, (b) *x* = 4 mm, and (c) *x* = 7 mm. (**d**) Distance between final position of beads at different flow velocities and magnet positions in simulation and experiment. (**e**) The time of beads crossed the main channel with different conditions in simulation 1.

The trajectories of magnetic beads were observed through simulation1 (ESI Video S1). As is shown in Figure 2a–c, the distribution range of magnetic beads on the bottom edge increases from 62.89 μm to 775.53 μm at velocity of 120 μL/h (about 4.155 mm/s in the channel) with the magnet placed from position 1 to 3. This is likely the same for other velocities of fluid. The shortest distance between the termination of magnetic beads and origin (entrance of the main channel) is 491.58 ± 18.07 μm at the fluid velocity of 1.373 mm/s with a permanent magnet at position 1. When the magnet is located at position 3 and the velocity is 4.155 mm/s (120 μL/h), the beads reach the longest position 5070.17 ± 387.77 μm away from the entrance (Figure 2d). The crossing time of beads in the main channel is described in Figure 2e. The maximum time difference is only 1.37 s because of the limited width of the main channel. For most experiments with reaction time of minutes or hours, this difference of time can be ignored.

**Figure 3:**
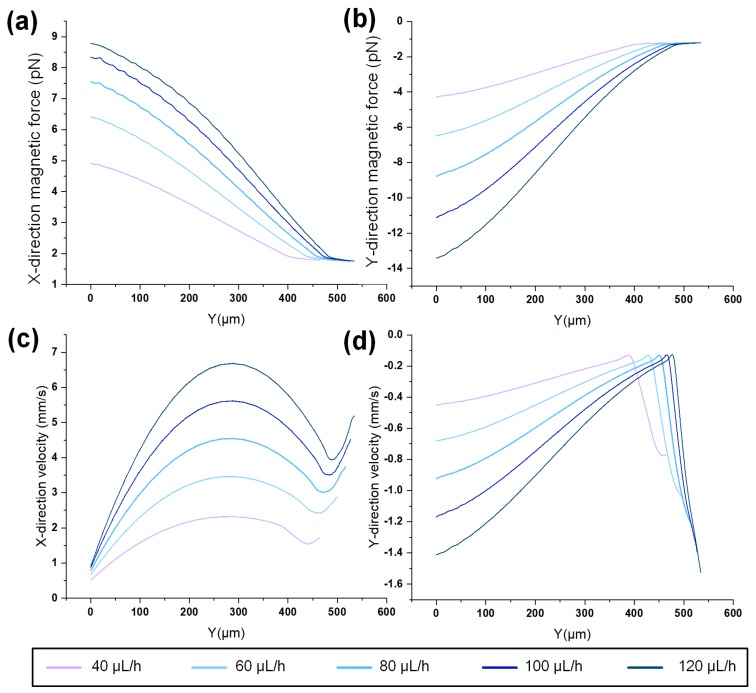
The magnetic force on the beads not impinging the branched wall in *x* (**a**) and *y* (**b**) direction with NdBFe magnet at position 3. The velocity of those beads in *x* (**c**) and *y* (**d**) direction increased a lot with the 𝑢_𝑓𝑙_ from 40 μL/h to 120 μL/h. The horizontal axis Y represents the vertical distance from the bead to the bottom edge.

During the process of simulation, we focused on the force, velocity, and residence position of the beads in the main channel (See Figure 3 and ESI Figures S3 and S4). However, these results are closely related to the motion state of beads in the branched channel. When the permanent magnet is at position 1, all the magnetic beads in the branched channel deflect to the Y direction under the influence of the large magnetic field, and they slop to the wall before entering the main channel (Figure 2a). As is shown in the velocity distribution of laminar flow in the channel (ESI Figure S5), the fluid velocity is very small at both sides of the channel. In addition, there is a certain interaction between beads and the wall. Thus, the wall-impingement beads slow down the velocity before entering the main channel and this initial velocity mostly depends on the position of the external magnet instead of the fluid velocity. Velocities of these beads at the entrance of the main channel are 0.97 ± 0.40 mm/s, 0.67 ± 0.12 mm/s, and 0.50 ± 0.39 mm/s in x direction, as well as −1.48 ± 0.09 mm/s, −0.42 ± 0.03 mm/s, and −0.25 ± 0.12 mm/s in y direction respectively with the magnet at position 1, 2, 3 (ESI Figure S4). The initial position of these wall-impingement beads is almost constant, which is about 400 μm from the bottom of the main channel in the y direction

When the magnet is at position 2, the distribution range of the arriving beads significantly increases from 64.14 μm to 291.75 μm with fluid velocity at 40 μL/h and 120 μL/h. This means the number of the beads collided with the wall of branched channel relies on the fluid velocity. When the magnet is at position 3, a majority of the beads deflect in the branched channel without touching the wall. In this condition, the initial velocity and position of the beads were determined by the fluid velocity (Figure 3). 

#### 4.1.2. Simulation of Main Channel Structure

The results of full-structure simulation comprehensively show the relationship between the trajectory of the bead and the position of permanent magnet. However, it takes a long time to calculate the movement of the beads in the branched channel, which has a limited impact on the study of bead movement in the main channel. As “ESI” Figures S6 and S7 shows, the magnetic flux density declines with the increase of the distance away from permanent magnet and it is likely the same for its gradient on the basis of the distribution of magnetic flux density. Therefore, when the magnetic beads are in the branched channel far from the permanent magnet, they are subjected to small magnetic force. When they move with the fluid into the main channel, the magnetic force imposed on them increases. That means we can focus more on the main channel to study the transport of magnetic beads. In addition, the wall-impingement beads entered the main channel with low initial velocities and constant positions according to the results of simulation 1. Therefore, the simulation structure can be simplified into the main channel as well as beads are released at a velocity of 0 mm/s from 400-micron height in y direction (ESI Figure S2). As a result, the final position of the magnetic beads can be quickly estimated under the varying conditions. These results are within the distribution range of beads compared to simulation 1 (Figure 2d).

#### 4.1.3. Experimental Verification of Magnetic Bead Trajectory

**Figure 4:**
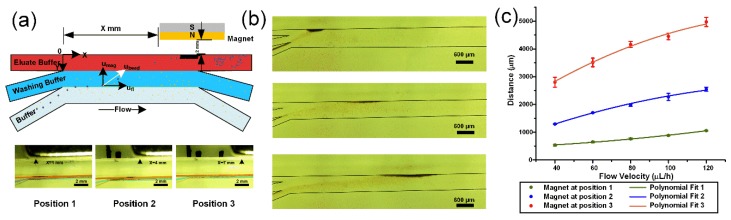
(**a**) The schematic and photos of magnetic beads purification of DNA on a chip. (**b**) All the magnetic beads were deflected to the wall in the upper picture (*x* = 1 mm, 𝑢_𝑓𝑙_ = 120 μL/h); x = 4 mm, 𝑢_𝑓𝑙_ = 120 μL/h in the middle picture; only a few of beads sloped to the wall before entering the main channel in the bottom picture (*x* = 7 mm, 𝑢_𝑓𝑙_ = 120 μL/h). (**c**) Fitting curve of distance between arriving position of beads and origin with the 𝑢_𝑓𝑙_ from 40 μL/h to 120 μL/h.

The influence of magnet positions and the fluid velocities on the trajectory of magnetic beads has also been verified by experiments. Figure 4b clearly shows that when the permanent magnet is at position 1, all the beads deflect to the wall of branched channel at the flow velocity of 120 μL/h. However, when the permanent magnet is at position 2 and 3, a small part of the beads deflects to the wall, and most of the beads get into the main channel after few deflections in the branched channel. After the magnetic beads reach the bottom edge of the main channel, they can stay there, which provides conditions for subsequent nucleic acid separation experiments. The distribution range of magnetic beads would enlarge after a certain quantity of magnetic beads arrive at the bottom edge (See Video S2). As the subsequent magnetic beads accumulate on the earlier beads, the fluid would push the upper magnetic beads forward, further broadening the distribution range. Therefore, it is difficult to make statistics on the whole distribution range. Here only the distance of beads nearest to the entrance has been counted with small input quantity of beads (Figure 2d and Figure 4c). By fitting a curve to data points, we can estimate the arrival distance of magnetic beads at other velocities (Figure 4c).

### 4.2. The Recovery Efficiency of DNA on A Chip

With this chip structure, automated nucleic acid recovery has been realized without liquid switch valve or other control module. According to previous simulation and experimental results, position 3 is chosen as the position of NdFeB permanent magnet. In this case, the magnetic beads can travel across the main channel at the maximum path to reach the bottom edge at different flow velocities. This allows the beads to remain for as long as possible in each buffer, allowing the DNA-carrying beads to be fully washed. At the same time, as the DNA-carrying beads finally remain at the bottom edge, DNA was continuously eluted while the new beads reach the bottom.

**Figure 5:**
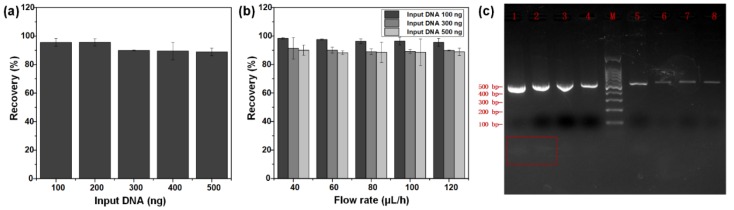
(**a**) The DNA recovery efficiency with input from 100 ng to 500 ng at the fluid velocity of 120 μL/h with a NdFeB magnet at position 3. (**b**) The trend of DNA recovery efficiency with the 𝑢_𝑓𝑙_ from 40 μL/h to 120 μL/h, with DNA input of 100 ng, 300 ng, and 500 ng. (**c**) 2% agarose gel electrophoresis image of PCR product and purified product. Lane 1 and lane 2, 507 bp PCR product; line 3 and line 4, product purified in the tube; line 5-8, product purified on the chip with the 𝑢_𝑓𝑙_ from 40 μL/h to 100 μL/h; line M: 100 bp DNA Ladder. Red box: diffused bands (extra primers).

The recovery efficiency of DNA can be calculated by comparing the known input amount of PCR products with the yield of recovered DNA on the chip, and the DNA was quantified via Qubit before and after the experiments. First, we explore the recovery efficiency of DNA with different input amounts. It shows that the recovery efficiency of DNA decreases with the increase of input samples from 100 ng to 500 ng at intervals of 100 ng at the flow rate of 120 μL/h (Figure 5a). The obvious drop (5.7%) of the DNA recovery efficiency could be observed at the input DNA of 300 ng. This rule is also applicable to the DNA recovery at other flow velocities. The reason for that possibly is the number and height of piled beads rise as the input of DNA increases, which means the subsequent beads could not be eluted completely. Most of the eluted space was occupied by the increased volume of previous beads, so the number of intermediate beads grows and the time for intermediate beads to be eluted decreases. Consequently, the elution time for the increased DNA-carrying beads declines when input of samples increases.

The Figure 5b also displays that the velocity of fluid has a slight impact on DNA recovery efficiency. When the input is 100 ng, the DNA recovery efficiency declined merely by 2.8% with the increase of flow rate from 40 μL/h (1.373 mm/s) to 120 μL/h (4.155 mm/s). Similarly, when the input sample size was set 300 ng and 500 ng, the DNA recovery efficiency drops more marginally (within 1.5% and 1.2%) with the change of flow rate. The maximum of recovery efficiency is 98.4% with the input of 100 ng and the flow rate of 40 μL/h.

DNA purity is evaluated by UV spectroscopic analysis and electrophoresis. The value of A260/A280 can show the purity of DNA, as double stranded DNA has the maximum value of absorbance at 260 nm and aromatic amino acids have the maximum value of absorbance at 280 nm. The DNA purity can be considered as good, when the A260/A280 ratio is 1.7~2.0. Table 1 shows that magnetic bead purification can obtain purified DNA regardless of purifying in a tube or on a chip, with the A260/A280 ratio between 1.8 and 2.0. In addition, 2% agarose gel electrophoresis image shows that only lane 1 and 2 have diffused bands that are shorter than 100 bp (See Figure 5c), which means the extra primers were removed in the process of magnetic bead purification on a chip.

## 5. Conclusions

In this paper, we demonstrate a branched microfluidic chip that can accomplish automated nucleic acid purification and isolation with an external permanent magnet. The computational simulation results of the beads movement are consistent with the experimental data, which brings convenience to other relative experiments on trajectory of magnetic bead. The simulation based on simplified structure focuses on the characteristics that magnetic beads have small velocities and constant positions at the entrance of main channel, which further reduces the time for simulation. In DNA recovery section, this microfluidic chip implements continuous purification of DNA in small amount. It is hopeful to increase the throughput of library preparation, as purification of DNA is one of the significant steps of library preparation. 

In previous research, a rotating permanent magnet and a circular structure of chip were utilized to actuate the beads inside the channels [26]. However, this method requires a large input and a rotating system, which is not suitable for experiments with rare samples. Future improvement to application of this module is combining it with droplet generation [31,32,33] and fusion module [34,35,36], to provide a fundament for further automated sample preparation. 

## Figures and Tables

**Table 1:** The A260/A280 ratio and A260/A230 ratio show the purification results of PCR product. The 𝑢_𝑓𝑙_ is 100 μL/h and DNA input is 100 ng when purifying DNA on chip.

Sample	260/280	260/230
**507 bp DNA purified in tube**	1.823	2.143
	1.853	2.242
	2.000	2.037
**507 bp DNA purified on chip**	1.960	2.133
	1.951	2.394
	1.973	2.237

## Supplementary Materials

Electronic Supplementary Information (ESI) The following are available online at https://www.mdpi.com/2072-666X/11/2/187/s1. Figure S1. (**a**) CAD diagram of complete structure of the chip. (**b**) Photograph of the PDMS chip filled with blue and red ink. Figure S2. (**a**) Schematic of simulation of complete structure (simulation 1). (**b**) Schematic of simulation of main channel (simulation 2). Table S1. Relevant parameters for COMSOL simulation. Figure S3. X-direction (**a**–**c**) and y-direction (**d**–**f**) magnetic force on the beads crossing the main channel with varying fluid velocities and the NdFeB magnet at different positions: (a) and (d) position 1, (b) and (e) position 2, (c) and (d) position 3. The data for the beads deflected to the wall of branched channel are selected, obtained from Comsol 5.3a. Figure S4. X-direction (**a**–**c**) and y-direction (**d**–**f**) velocities of beads crossing the main channel with varying fluid velocities and the NdFeB magnet at different positions: (a) and (d) position 1, (b) and (e) position 2, (c) and (d) position 3. The data for the beads deflected to the wall of branched channel are selected, obtained from Comsol 5.3a. Figure S5. (**a**) Distribution of laminar fluid velocity of the whole chip. (**b**) Distribution of laminar fluid velocity in the branched channel. Figure S6. Simulation results of magnetic flux density of a 1 × 1 × 2 cm^3^ NdFeB magnet (Comsol 5.3a). Figure S7. simulation of magnetic flux density across y direction of the main channel at different position in the x-direction (Comsol 5.3a). A 1 × 1 × 2 cm^3^ NdFeB magnet was placed at different distances from the origin: (**a**) 1 mm, (**b**) 4 mm, and (**c**) 7 mm. Video S1. Trajectory of beads in simulation1. Video S2. The movement of beads on the chip.
