# Supplementary material for: Continuous Microfluidic Purification of DNA Using Magnetophoresis
Source: Micromachines (Basel). 2020 Feb 11;11(2):187. doi: 10.3390/mi11020187 (PMC7074667; doi:10.3390/mi11020187)
Supplement: Supplementary file 1 [file micromachines-11-00187-s001.pdf]

micromachines

Electronic Supplementary Information (ESI)

Continuous Microfluidic Purification of DNA Using Magnetophoresis

Ying Xu [1], Zhen Zhang [1], Zhen Su [1], Xiaoxiang Zhou [1], Xiaoming Han [1], Quanjun Liu [2,*]

[1] State Key Laboratory of Bioelectronics, School of Biological Science and Medical Engineering, Southeast University, Nanjing 210096, China; 220174594@seu.edu.cn (Y.X.); 220181842@seu.edu.cn (Z.Z.); 220174564@seu.edu.cn (Z.S.); 230189166@seu.edu.cn (X.Z.); 220171812@seu.edu.cn (X.H.)

[2] Shenzhen Research Institute of Southeast University, Shenzhen Virtual University Park, Shenzhen 518000, China

* Correspondence: 101005442@seu.edu.cn; Tel: +86-25-83793283

Received: 7 January 2020; Accepted: 7 February 2020; Published: date

<u>Content</u>

The structure of the chip 2

Schematic of simulation 2

Parameters for COMSOL simulation 2

Results of simulations 2

Video S1-Trajectoty of beads in simulation1 .... **Error! Bookmark not defined.**

Video S2-The movement of beads on the chip.............................. **Error! Bookmark not defined.**

The structure of the chip

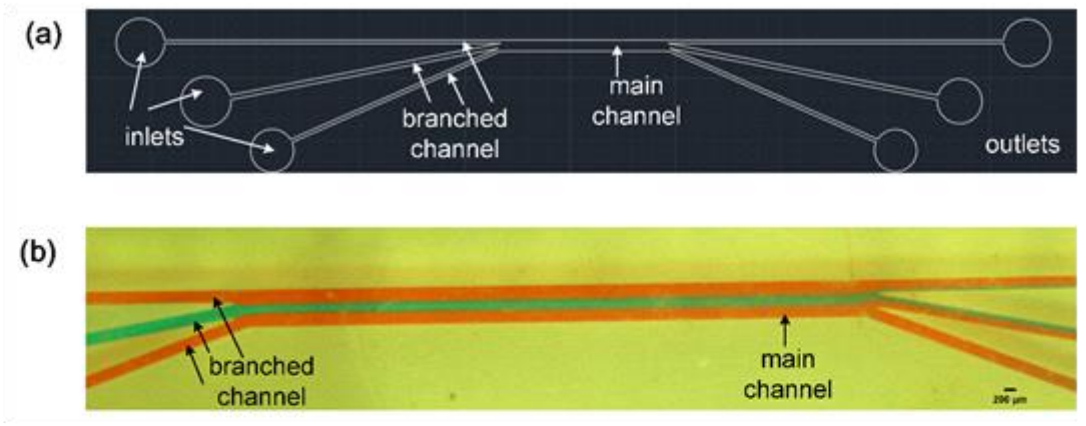

Figure S1. (**a**) CAD diagram of complete structure of the chip. (**b**) Photograph of the PDMS chip filled with blue and red ink.

Schematic of simulation

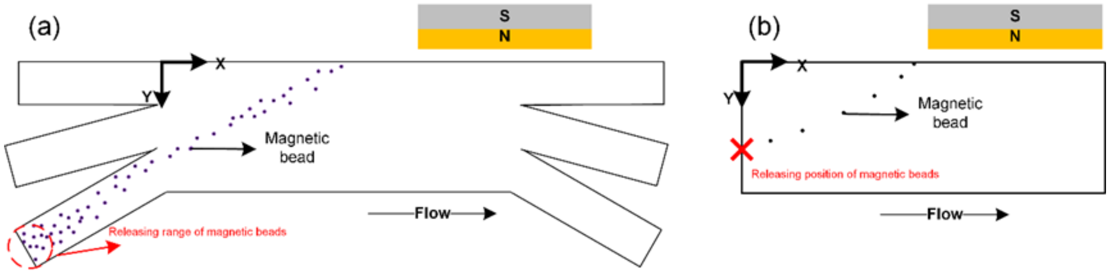

Figure S2. (**a**) Schematic of simulation of complete structure (simulation 1). (**b**) Schematic of simulation of main channel (simulation 2).

Parameters for COMSOL simulation

Table S1. Relevant parameters for COMSOL simulation.

Parameter	Value
Remanence of permanent magnet (Br)	1.45 T
Dynamic viscosity of fluid (η)	1.01×10^{-3} Pa·s
Radius of beads (r)	0.5 μm
Density of beads	1400 kg/m^3
Density of fluid	1000 kg/m^3
Magnetic susceptibility of beads (χ)	3
Permeability of free space (μ_0)	$4\pi \times 10^{-7}$ N/A^2

Results of Simulation

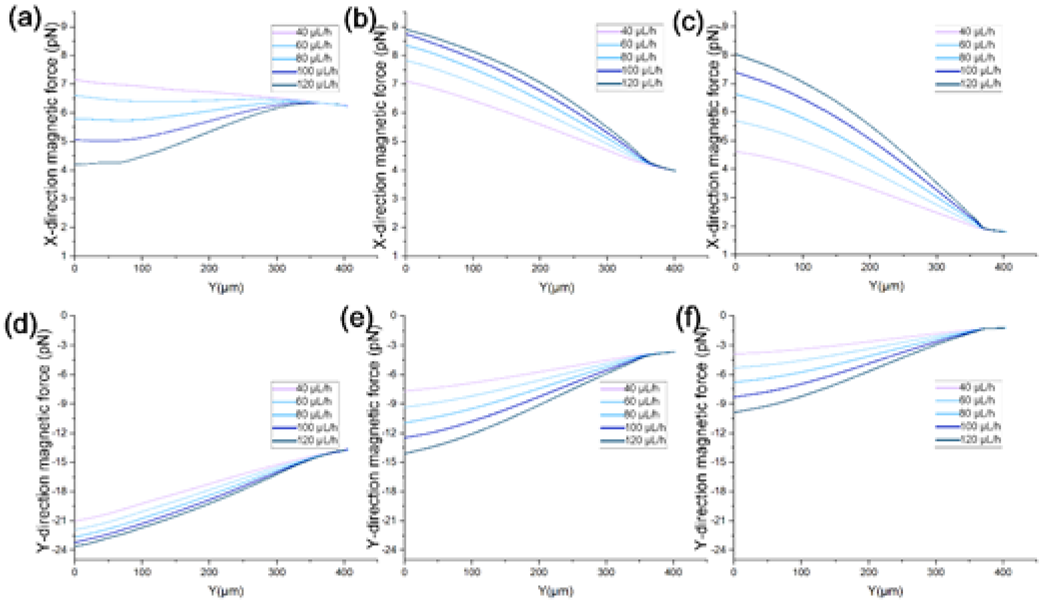

Figure S3. X-direction (**a–c**) and y-direction (**d–f**) magnetic force on the beads crossing the main channel with varying fluid velocities and the NdFeB magnet at different positions: (a) and (d) position 1, (b) and (e) position 2, (c) and (d) position 3. The data for the beads deflected to the wall of branched channel are selected, obtained from Comsol 5.3a.

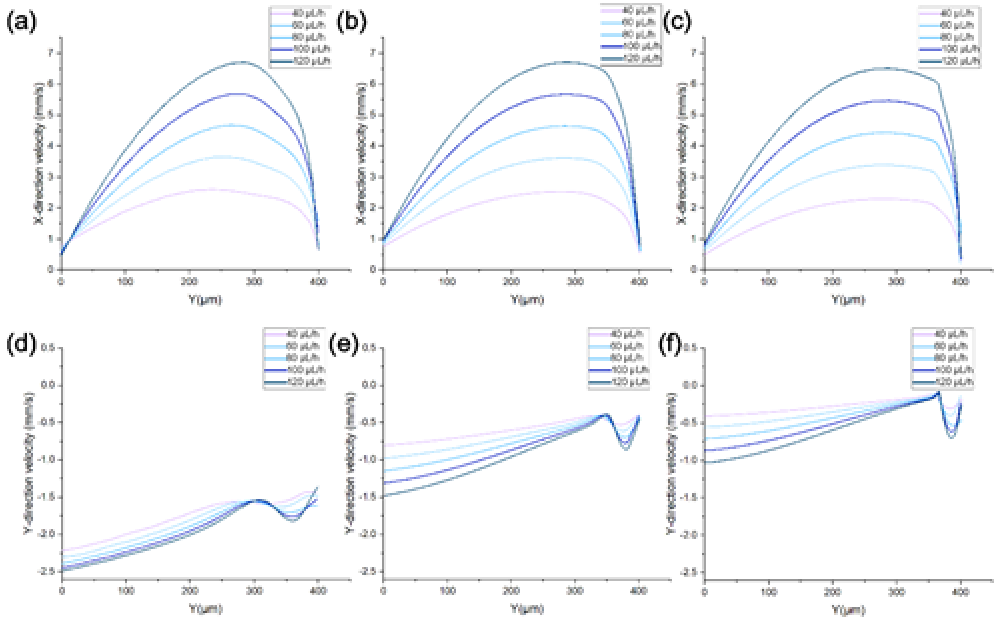

Figure S4. X-direction (**a–c**) and y-direction (**d–f**) velocities of beads crossing the main channel with varying fluid velocities and the NdFeB magnet at different positions: (a) and (d) position 1, (b) and (e) position 2, (c) and (d) position 3. The data for the beads deflected to the wall of branched channel are selected, obtained from Comsol 5.3a.

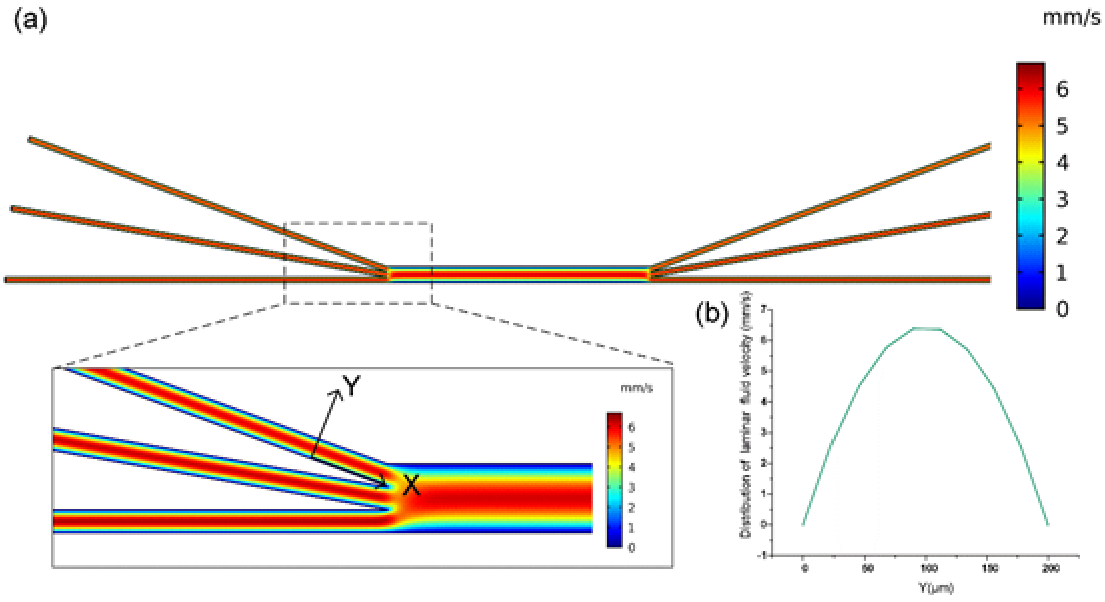

Figure S5. (**a**) Distribution of laminar fluid velocity of the whole chip. (**b**) Distribution of laminar fluid velocity in the branched channel.

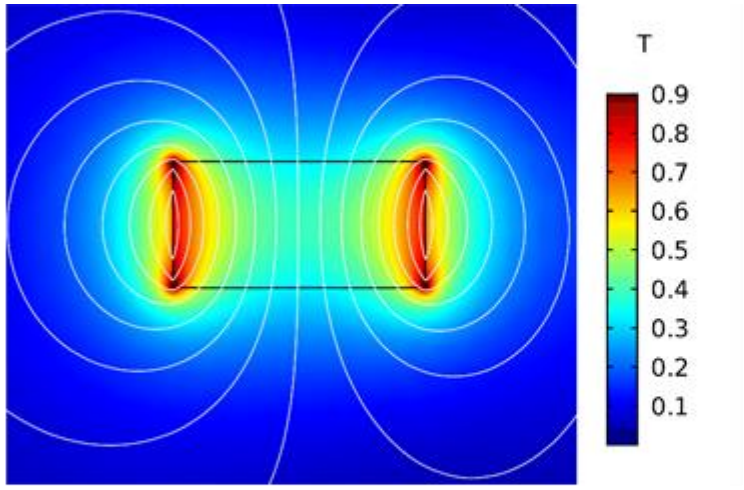

Figure S6. Simulation results of magnetic flux density of a 1 × 1 × 2 cm3 NdFeB magnet (Comsol 5.3a).

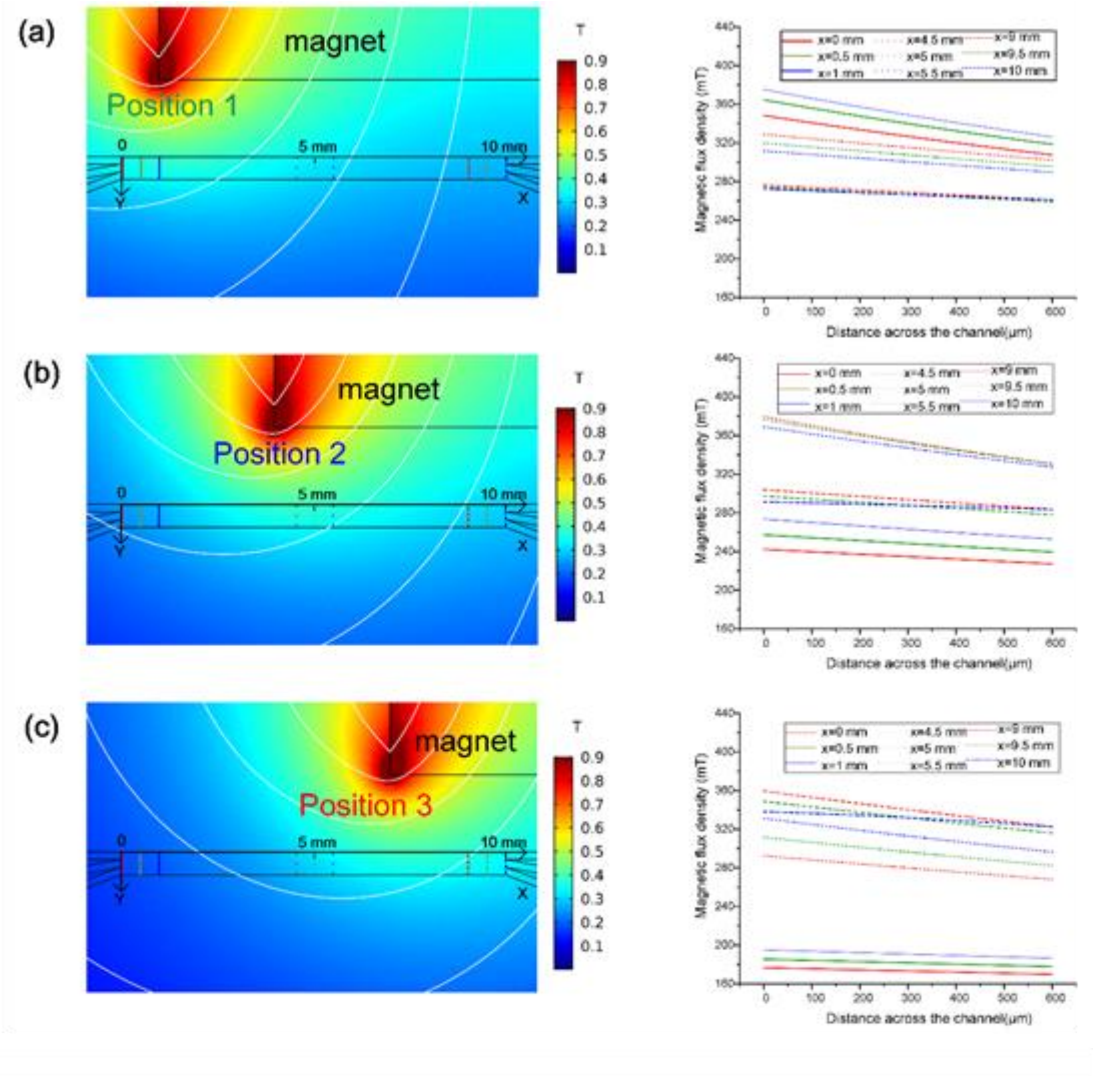

Figure S7. Simulation of magnetic flux density across y direction of the main channel at different position in the x-direction (Comsol 5.3a). A 1 × 1 × 2 cm^3 NdFeB magnet was placed at different distances from the origin: (**a**) 1 mm, (**b**) 4 mm, and (**c**) 7 mm.

Video S1-Trajectoty of beads in simulation1

Video S1 shows the movement of the beads with a magnet at position 3 and u_{fl} = 4.155 mm/s.

Video S2-The movement of beads on the chip

Video S2 shows the movement of the beads with a magnet at position 1 and varying fluid velocity from 40 μL/h to 120 μL/h.
